# Hydride state accumulation in native [FeFe]-hydrogenase with the physiological reductant H_2_ supports its catalytic relevance†

**DOI:** 10.1039/d2cc00671e

**Published:** 2022-05-30

**Authors:** Moritz Senger, Tobias Kernmayr, Marco Lorenzi, Holly J. Redman, Gustav Berggren

**Affiliations:** Department of Chemistry, Physical Chemistry, Uppsala University 75120 Uppsala Sweden moritz.senger@kemi.uu.se; Department of Chemistry, Molecular Biomimetics, Uppsala University 75120 Uppsala Sweden gustav.berggren@kemi.uu.se

## Abstract

Small molecules in solution may interfere with mechanistic investigations, as they can affect the stability of catalytic states and produce off-cycle states that can be mistaken for catalytically relevant species. Here we show that the hydride state (H_hyd_), a proposed central intermediate in the catalytic cycle of [FeFe]-hydrogenase, can be formed in wild-type [FeFe]-hydrogenases treated with H_2_ in absence of other, non-biological, reductants. Moreover, we reveal a new state with unclear role in catalysis induced by common low pH buffers.

## Abstract 2

Studies of enzymatic catalysis often rely on non-biological reagents, which may affect catalytic intermediates and produce off-cycle states. Here the influence of buffer and reductant on key intermediates of [FeFe]-hydrogenase are explored.

## Graphical abstract

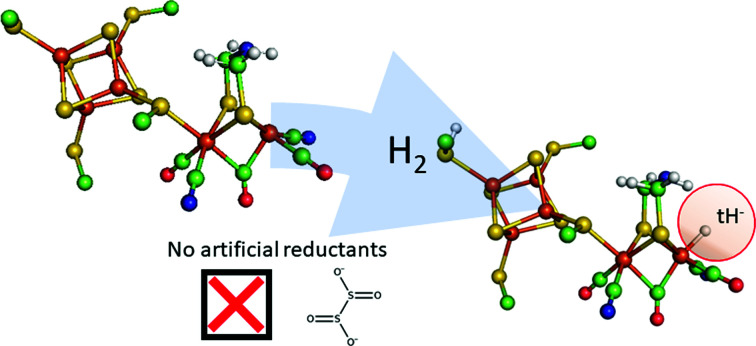

Hydrogenases are redox enzymes catalysing molecular hydrogen (H_2_) uptake and production. The high H_2_ evolution frequency of prototypical [FeFe]-hydrogenases motivates basic research on the reaction mechanism and inspires catalyst design for a hydrogen economy.^1–3^ In these enzymes, hydrogen catalysis takes place at a hexa-iron cofactor, called the H-cluster. It is composed of a [4Fe4S]-cluster linked *via* a cysteine residue to a unique diiron site ([2Fe]) that binds two cyanide (CN^−^) and three carbonyl (CO) ligands. These ligands serve as intrinsic probes sensitive to infrared spectroscopy and report on reduction and protonation events during catalysis. The diiron site is bridged by an azadithiolate ligand (^−^SCH_2_NHCH_2_S^−^, ADT) that is proposed to shuttle protons *via* its amine group to the apical vacancy, the site of hydrogen catalysis, located at the Fe ion (Fe_d_) distal to the [4Fe4S] cluster (Fig. 1). The most studied group of [FeFe]-hydrogenases (Group A) transfers protons to the active site *via* a conserved Proton Transfer Pathway (PTP) composed of amino acid residues and water molecules.^4–6^ Recently described [FeFe]-hydrogenases in group D lack this PTP.^7,8^ Several redox and protonation states have been characterised and proposed as catalytic intermediates.^1,2^ The starting point of catalysis is the oxidised state H_ox_. Under inert atmosphere conditions at low pH values and in presence of chemical reductant a blue-shifted variant of H_ox_ is formed, commonly denoted H_ox_H.^9,10^ One electron reduction of H_ox_ results in population of two singly reduced states: H_red’_ or H_red_. H_red’_ features a reduced [4Fe4S] cluster while the diiron site is still in an oxidised configuration. Formation of the diiron site reduced state H_red_ is coupled to a proton uptake event, although the site of protonation is under discussion.^11,12^ A second reduction step yields the super-reduced state (H_sred_) or a hydride state (H_hyd_). H_sred_ is characterised by a reduction of both the [4Fe4S] cluster and the diiron site.^13^ In contrast, H_hyd_ features a terminal hydride at Fe_d_, yielding a formally oxidized diiron site (Fig. 1).^14–16^ As the last step in hydrogen turnover, the terminal hydride of H_hyd_ is proposed to combine with a proton delivered *via* the PTP to generate molecular hydrogen.^1–3^ A hydride state similar to H_hyd_ but with an additionally protonated ADT bridge has been proposed as the last intermediate in hydrogen evolution.^17–20^

**Fig. 1:**
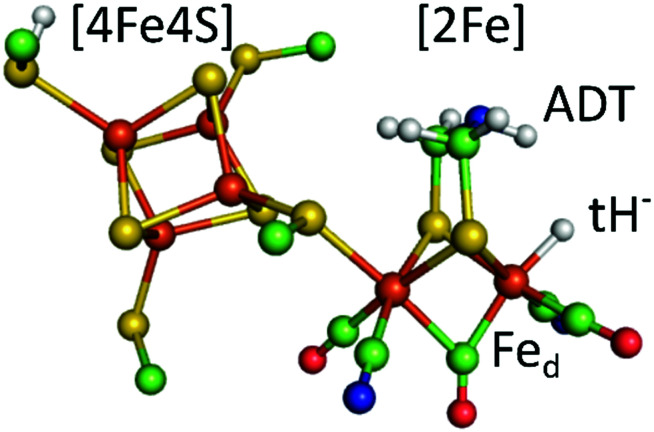
The calculated hydride state structure of the H-cluster in [FeFe]-hydrogenases. The H-cluster consists of a protein bound [4Fe4S] cluster that is covalently linked *via* a cysteine thiolate ligand to the diiron site ([2Fe]), further ligated by three carbonyl (CO) and two cyanide (CN^−^) ligands. A terminal hydride (tH^−^) at the distal iron ion is proposed to combine with a proton delivered by the ADT ligand to yield molecular hydrogen in the last step of hydrogen catalysis. Colour code: orange-iron, yellow-sulphur, green-carbon, red-oxygen, blue-nitrogen, white-hydrogen. The structure is drawn after the PDB coordinates 4XDC optimized for the Hhyd state by DFT calculations. (ref. 33).

A terminal-hydride state of the H-cluster, considered to reflect the H_hyd_ state, can be stabilised by impairing the enzyme's catalytic function *via* amino acid variations in the PTP or cofactor alteration. This has enabled its characterization using a range of techniques, including NMR, NRVS, Mössbauer, EPR and FTIR spectroscopy.^14–16,18,21,22^ In native, fully functional, [FeFe]-hydrogenases the accumulation of a highly similar H_hyd_ state is commonly achieved by exposure to H_2_ at low pH values in the presence of the non-physiological reductant sodium dithionite (NaDT, Table S1, ESI†) (with exceptions^19,22–24^).^16,17,19,22,25,26^ Based on chronoamperometry experiments it was recently proposed that [FeFe]-hydrogenases are inhibited by NaDT at low pH values (or rather SO_2_, one of its oxidized by-products), *i.e.* conditions similar to H_hyd_ accumulation.^27^ Considering its in-depth spectroscopic characterization, and proposed central importance in the catalytic cycle, the possibility that the to-date characterized H_hyd_-state reflects an inhibited “artefact-state” would represent a significant setback in our mechanistic understanding of [FeFe]-hydrogenase.

Here, we selectively enrich the double reduced states, H_hyd_ and H_sred_, by exposure to molecular hydrogen and follow the respective absorbance changes of cofactor ligand bands by Attenuated Total Reflection Fourier-transform Infrared (ATR-FTIR) Spectroscopy. Data is collected at different pH values and buffers. This study reveals that small carboxylic acids, often used in low pH buffers in enzyme electrochemistry experiments, are non-innocent in formation of a new H-cluster species similar to H_ox_. At present it is not clear if this is an artefactual off-cycle state, or in fact a critical intermediate that previously have escaped detection. Moreover, we show the accumulation of H_hyd_ in native [FeFe]-hydrogenase, HydA1 from Chlamydomonas reinhardtii, at mildly basic and low pH values regardless of buffer choice and more importantly in the absence of NaDT. Our findings are well in accordance with the previously characterized H_hyd_-state as a reaction intermediate rather than an experimental artefact.

A solution of the HydA1 [FeFe]-hydrogenase was applied to the crystal surface of the ATR-FTIR setup, following removal of any NaDT contaminations by chromatography and buffer exchange. The enzyme solution was subsequently dried and rehydrated under N_2_ atmosphere to form an auto-oxidised protein film.^28,29^ The hydrogenase adopted the expected oxidised active-ready state H_ox_, and was subsequently exposed to H_2_ gas. The resulting difference spectra are displayed in Fig. 2. As a consequence of H_2_ uptake, a mixture of the hydride state H_hyd_ (blue positive bands, 2081, 2066, 1979, 1960, 1860 cm^−1^) and the super-reduced state H_sred_ (red positive bands, 2070, 2025, 1954, 1918, 1882 cm^−1^) was populated while the oxidised state H_ox_ (grey negative bands, 2089, 2071, 1965, 1940, 1802 cm^−1^) was depopulated. This holds true for different pH values, in a range of different buffers. Fig. 2 displays the ATR-FTIR difference spectra of HydA1 samples adjusted to pH 8 or pH 4 and subsequently exposed to H_2_ (absolute spectra and pH adjustment in Fig. S1, ESI†). In each spectrum the signature of H_hyd_ (blue positive bands) is clearly detectable. A larger fraction of H_sred_ is formed at pH 8 (100 mM Tris buffer) while a larger fraction of H_hyd_ is populated at pH 4 (100 mM propionate buffer), in both cases a concomitant loss of H_ox_ is observed in contrast to earlier results where a depopulation of H_ox_H was observed at pH 4 due to the presence of NaDT.^9,10,16^ However, the preference for H_hyd_ accumulation at low pH is clearly independent of the presence of NaDT. As H_hyd_ and H_sred_ are generally considered to reflect two different tautomers, their relative stability as a function of pH value is arguably a consequence of the protonation state of the H-cluster surroundings. The reaction of auto-oxidised HydA1 films with H_2_ gas proceeds within one-two seconds while auto-oxidation, observed when H_2_ was replaced by N_2_, occurs over 10–20 seconds (compare Fig. S2, ESI†). Both kinetics report indirectly on the gas accessibility and hydration of the protein film in the ATR-FTIR setup. Dehydrated [FeFe]-hydrogenase samples were reported to be protected from exposure to gases.^30^

**Fig. 2:**
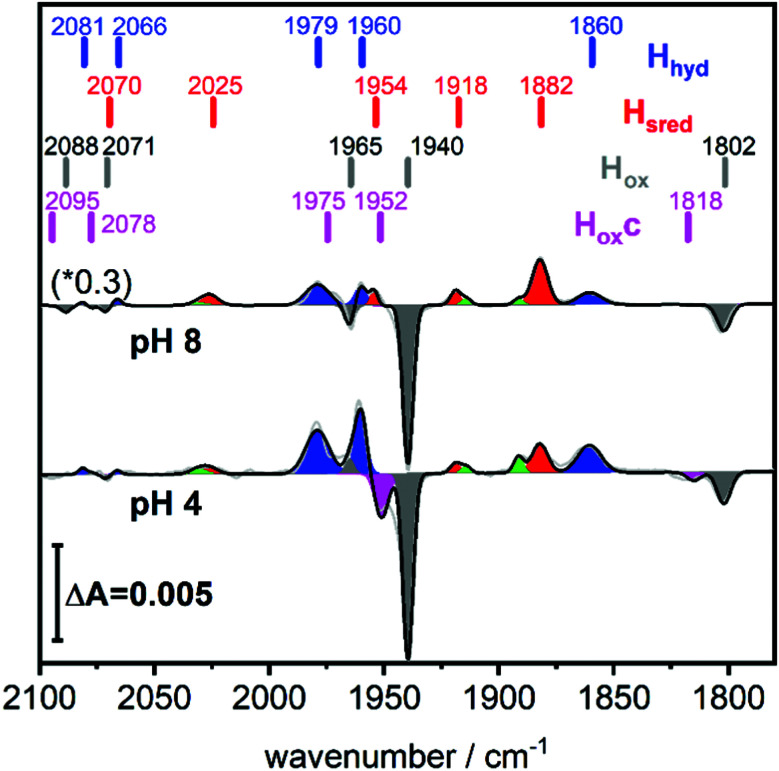
Hydrogen uptake induced ATR-FTIR difference spectra of the CO/CN ligands of HydA1 Upon exposure to H_2_ the oxidised state H_ox_ (grey negative bands) is depopulated while double reduced redox states H_sred_ (red positive bands) and H_hyd_ (blue positive bands) accumulate. For both pH values, pH 8 (top, 100 mM Tris buffer, scaled by 0.3) and pH 4 (bottom, 100 mM propionate buffer), the formation of H_hyd_ (blue) is observed in the absence of NaDT. Magenta negative bands at 1952 and 1815 cm^−1^ indicate the newly observed, unknown H-cluster species denoted H_ox_c. Green bands belong to H_red_. The band positions are indicated by bars.

Fig. 2 (bottom) shows that H_2_ exposure causes H_hyd_ accumulation in the absence of NaDT preferentially at low pH values (compare Fig. 2 top). This trend had been reported by several groups before, however always involving NaDT.^16,17,19,25^ Apart from the recent proposed binding of NaDT (or one of its oxidized products) to the H-cluster, protonation has been suggested to stabilize the reduction at the [4Fe4S] cluster for H_red’_^9,10,31^ and a similar stabilisation effect was reported for H_hyd_ from both purely computational and spectroscopic approaches.^32,33^ Our findings verify that NaDT is not required to stabilize H_hyd_. The observed pH dependent shift between H_sred_ and H_hyd_ instead supports a model of a protonatable site, either near the reduced [4Fe4S] cluster as previously proposed,^3,31,34^ or *e.g.* in the PTP. Residual traces of NaDT have been shown to lead to population of H_ox_H (2089, 2079, 1970, 1945, 1810 cm^−1^),^27^ which is absent in our spectra. The negative bands in the pH 4 difference spectrum (Fig. 2 bottom) at 1952 and 1815 cm^−1^ belong to an unknown band pattern significantly different from H_ox_H (full spectroscopic signature Fig. 3), and we denote this new state as H_ox_c.

**Fig. 3:**
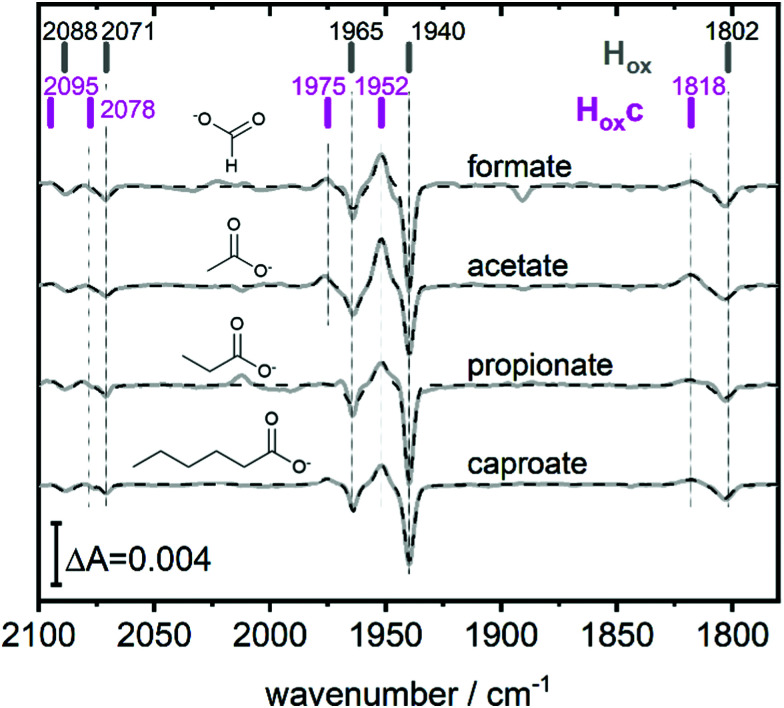
The full spectroscopic signature of H_ox_c. Difference spectra highlighting the full band pattern of H_ox_c (positive bands) for transitions from pH 8 (10 mM Tris, negative bands) to pH 4 with, from top to bottom, 100 mM formate, 100 mM acetate, 100 mM propionate and 100 mM caproate buffer. Data presented in grey and fit in dashed black. The band positions of H_ox_ and H_ox_c are indicated by bars. Acetate and propionate spectrum scaled by 2 and 0.5 respectively.

This blue shifted H_ox_ population was observable with pH 4 buffers composed of monocarboxylic acids, *i.e.* formic acid (p*K*_a_1__ = 3.8), acetic acid (p*K*_a_ = 4.8), propionic acid (p*K*_a_1__ = 4.9) and caproic acid (p*K*_a_1__ = 4.9) (Fig. 3). Conversely, di- and tricarboxylic acids such as succinic acid (p*K*_a_1__ = 4.2), oxalic acid (p*K*_a_2__ = 4.14) or citric acid (p*K*_a_1__ = 3.1) did not induce the new species (Fig. S3, ESI†). Thus, stabilizations of H_ox_c appear to be determined by the number of the carboxylic acid groups, rather than molecular weight of the acid.

Compared to H_ox_ the overall band pattern of H_ox_c is retained and in analogy to H_red’_ (red shifted) and H_ox_H (blue shifted) we propose that the H-cluster geometry and electronic structure is highly similar. Moreover, the requirement for low pH in forming the state implies that the buffer molecule interacts in its protonated, uncharged, form (Fig. S4, ESI†). A complete characterisation of this new H-cluster state is beyond the scope of the current study, but we note that it could be of high relevance in particular as *e.g.* acetic acid (acetate) is commonly employed in protein film electrochemistry studies of [FeFe]-hydrogenase.^27,35–45^ Formaldehyde has previously been observed to act as an inhibitor of the H-cluster under reducing conditions.^46,47^ In the latter case, binding of formaldehyde on the [2Fe] subsite *via* a Fe–C bond was proposed from a combination of ENDOR spectroscopy and DFT calculations,^48^ and further supported by studies of model complexes.^49^ Whether a similar binding model can be applied also to carboxylic acids remains to be verified. The extent of the new state (H_ox_c) formed in electrochemistry setups, its role in catalysis and its implication on catalytic currents, especially at low pH, remain unclear. In contrast to the H_ox_ state, the same H_hyd_ state forms regardless of the nature of the buffer, rendering a stabilizing role of the buffer or other additives unlikely (Fig. 2 and Fig. S1, ESI†). Our data shows clearly that H_hyd_ can be accumulated in the absence of NaDT and without introducing loss-of-function mutations. In closing, this underscores the flat energy landscape of the H-cluster during catalysis, and it is tempting to conclude that the previously characterized H_hyd_ state indeed represents one of the final intermediates in hydrogen evolution catalysis of [FeFe]-hydrogenases.

Investigation by M. S. and T. K., M. L. (enzyme purification), H. J. R. (cofactor synthesis), writing (original draft) by M. S., writing (review & editing) M. S., T. K., M. L., H. J. R. and G. B.

The authors would like to thank Leif Hammarström for critically reviewing the manuscript. Open access funding was provided by Uppsala University. The European Research Council (ERC, to GB, Contract No. 714102) as well as the European Union's Horizon 2020 research and innovation program (Marie Skłodowska Curie Grant No. 897555 to M.S.) are gratefully acknowledged for funding.

## Conflicts of interest

There are no conflicts to declare.

## Supplementary Material

CC-058-D2CC00671E-s001
