# Supplementary material for: Hydride state accumulation in native [FeFe]-hydrogenase with the physiological reductant H2 supports its catalytic relevance
Source: Chem Commun (Camb). 2022 May 30;58(51):7184–7. doi: 10.1039/d2cc00671e (PMC9219605; doi:10.1039/d2cc00671e)
Supplement: CC-058-D2CC00671E-s001 [file CC-058-D2CC00671E-s001.pdf]

Electronic Supplementary Material (ESI) for ChemComm.
This journal is © The Royal Society of Chemistry 2022

Supporting Information

Hydride state accumulation in native [FeFe]-hydrogenase with the physiological reductant H_2 supports its catalytic relevance†

Moritz Senger,*[a] Tobias Kernmayr,[b, ††] Marco Lorenzi,[b] Holly J. Redman[b] and Gustav Berggren*[b]

a) Department of Chemistry, Physical Chemistry, Uppsala University, 75120 Uppsala, Sweden

b) Department of Chemistry, Molecular Biomimetics, Uppsala University, 75120 Uppsala, Sweden

††) Current address: Department of Chemistry, Ludwig-Maximilians-Universität München, 81377 München, Germany

*To whom correspondence should be addressed

moritz.senger@kemi.uu.se and gustav.berggren@kemi.uu.se

Contents:

Materials and Methods

Table S1 Overview of NaDT concentrations involved in characterization of H_{hyd} in previous studies.

Figure S1 Absolute and difference ATR-FTIR spectra of HydA1 during the pH 4 adjustment and under H_2 and N_2.

Figure S2 Gas interaction kinetics of HydA1 at pH 4. Plotted is the difference in peak area associated with each redox state over time.

Figure S3 Absolute ATR-FTIR spectra of HydA1 exposed to different buffers.

Figure S4 Absolute and difference ATR-FTIR spectra of HydA1 equilibrated with mixed buffer at pH 8 and pH 4.

Figure S5 Absolute ATR-FTIR spectra of HydA1 equilibrated with mixed buffer at pH 8 (10 mM Tris buffer) and pH 4 with 100 mM capronate buffer.

Supporting References

Materials and Methods:

Protein purification and the absence of NaDT *Chlamydomonas reinhardtii* HydA1 was expressed, purified and reconstituted with minor modifications as previously reported.[1, 2] The codon-optimized gene coding for *Cr*HydA1 was cloned in frame with a Strep-tag II on a pET11a vector by GeneScript, that was then used BL21(DE3) E. coli strain. The enzyme was aerobically expressed in its *apo*-form and purified using pre-packed StrepTrap columns on an Äkta-Ready system (Cytiva). After semi-enzymatic [4Fe4S] cluster reconstitution, the enzyme was matured using a $[Fe_2(adt)(CO)_4(CN)_2]^{2-}$ (adt = azadithiolate, $^-SCH_2NHCH_2S^-$) synthetic mimic as follows: a mixture containing 100uM reconstituted *Cr*HydA1, 600uM $Fe_2(adt)(CO)_4(CN)_2{}^{2-}$, 2mM sodium dithionite was prepared in a 100mM Tris/HCl pH 8, 200mM KCl buffer; the mixture was incubated for 90 minutes at room temperature and excess mimic and dithionite were removed using a PD10 desalting column, pre-equilibrated with a 100mM Tris/HCl pH 8, 200mM KCl buffer. To ensure complete removal of residual dithionite, the buffer was further exchanged to 10mM Tris/HCl pH 8 via four concentration and dilution cycles using Amicon® Ultra 0.5 Centrifugal Filter Units (Millipore), as recommended by the manufacturer.

ATR-FTIR spectroscopy 1µl enzyme solution (1mM CrHydA1) in 10 mM Tris buffer (pH 8) was deposited on the ATR crystal in the anaerobic atmosphere of a Braun Glove box. The ATR unit (BioRadII from Harrick) was sealed with a custom build PEEK cell that allowed for gas exchange and illumination (inspired by Stripp et al.[3, 4]) mounted in a FTIR spectrometer (Vertex V70v, Bruker). The sample was dried under 100% nitrogen gas and rehydrated with a humidified aerosol (100 mM Tris-HCl (pH 8) or 100 mM propionate/acetate/formate/capoate buffer (pH 4)) as described before[5]. Spectra were recorded with 2 cm^{-1} resolution, a scanner velocity of 80 Hz and averaged of varying number of scans (mostly 1000 Scans). All measurements were performed at ambient conditions (room temperature and pressure, hydrated enzyme films).

Table S1: Overview of NaDT concentrations involved in characterization of H_{hyd} in previous studies.

NaDT concentrations	[FeFe]-hydrogenase sample	Reference	Ref. number in main text
5 - 178 mM	WT/C169S (HydA1)	Mulder et al. 2017 JACS[6]	Ref. 14
2 mM	ODT (HydA1) [a]	Reijerse et al. 2017 JACS[7]	Ref. 15
2 mM	WT/ODT/C169A (HydA1), WT/E279A(*Cp*I), WT (DdH)	Winkler et al. 2017 Nat Commun[8]	Ref. 16
10-20-fold molar eq. relative to Fe content	WT (HydA1)	Mulder et al. 2013 JACS[9]	Ref.17
5 - 50 mM	WT/C169S (HydA1), C289S (*Ca*I)	Mulder et al. 2014 JACS[10, b]	Ref. 18
0 - 100 mM	WT (HydA1 in *E.coli* cells)	Meszaros et al. 2020 ChemSci[11]	Ref. 19
2 - 10 mM	C169S (HydA1)	Knoerzer et al 2012 J Biol Chem[12]	Ref. 21
10 - 100 mM	WT (HydA1) WT/C298S (*Ca*I)	Ratzloff et al. 2018 JACS[13, c]	Ref. 22
2 mM, personal communication	WT (HydA1)	Lorent et al. 2020 JACS[14]	Ref. 25
100 mM	WT (HydA1/DdH)	Pelmenschikov et al. JACS 2017[15]	Ref. 26

[a] ODT denotes a form of the enzyme matured with $[Fe_2(odt)(CO)_4(CN)_2]^{2-}$ (odt = oxadithiolate, $^-SCH_2OCH_2S^-$)

[b] FTIR and EPR spectra are reported for "auto oxidized" samples of the loss-of-function variant $CrHydA1^{C169S}$ where NaDT has been removed by gel filtration. Traces of a rhombic EPR signal attributable to the H_{hyd} state are visible in one "NaDT free" sample, no signal attributable to H_{hyd} is observed by FTIR in the absence of NaDT.

[C] FTIR spectra are reported for H_2 and D_2 treated auto-oxidized samples of WT CrHydA1 and CaI. Traces of a partial H_{hyd} FTIR signature are visible in the spectra, collected at pH and pD = 8.

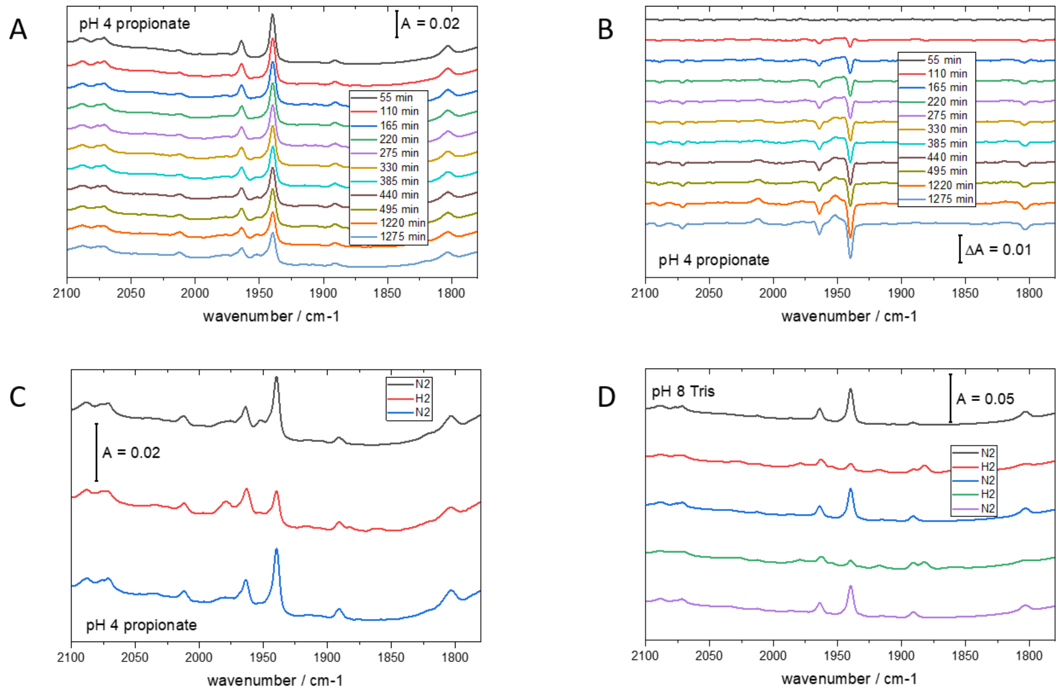

Figure S1 Absolute and difference ATR-FTIR spectra of HydA1 during the pH 4 adjustment and under H_2 and N_2. (A) Absolute spectra of HydA1 (10mM Tris pH 8) exposed to a 100 mM propionate buffer (pH 4) via the aerosol. Formation of the new species $H_{ox}c$ is followed over time. **(B)** Difference spectra of the same process as in (A). **(C)** Absolute spectra of HydA1 equilibrated at pH 4 (100mM propionate, compare (A) and (B)) exposed to H_2 and N_2. **(D)** Absolute spectra of HydA1 at pH 8 (100 mM Tris) exposed to H_2 and N_2. All spectra read from top to bottom. Note that a small population of H_{ox}-CO is present in all spectra (small peak at 2013 cm^{-1}). Over the time course of 20 h its population changes only slightly (e.g. compare (B)).

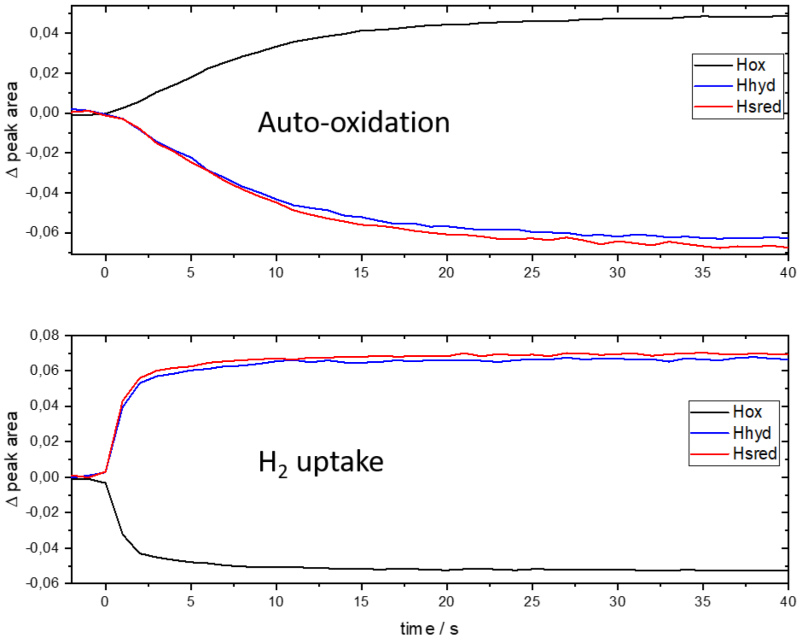

Figure S2 Gas interaction kinetics of HydA1 at pH 4. Plotted is the difference in peak area associated with each redox state over time. H_{ox} is scaled by 0.5. **top:** The sample exposed to N_2 after being equilibrated under H_2 (compare Fig.S1 C) adjusts to the new gas atmosphere (auto-oxidation) within ca. 20 seconds. **bottom:** The sample exposed to H_2 after being equilibrated under N_2 (compare Fig.S1 C) adjusts to the new gas atmosphere (H_2 uptake) within ca. 2 seconds.

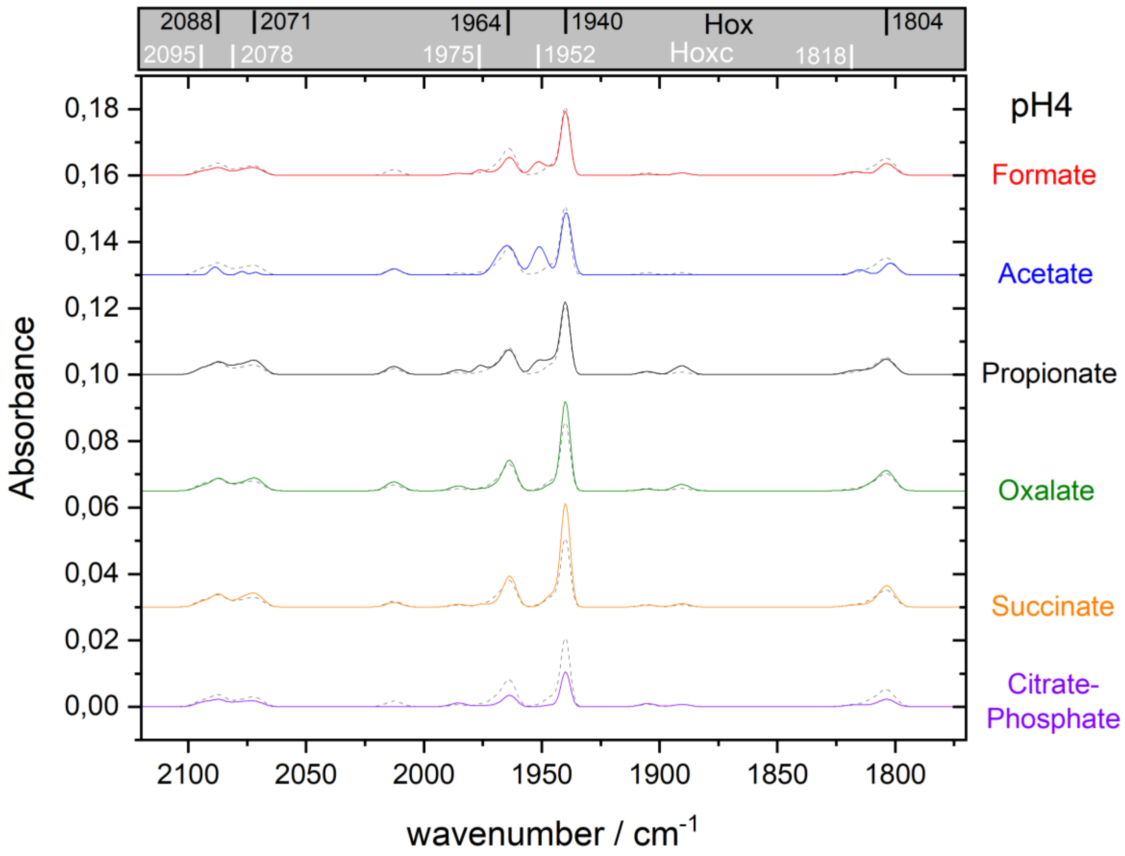

Figure S3 Absolute ATR-FTIR spectra of HydA1 exposed to different buffers. HydA1 exposed to different pH 4 buffers (100 mM) composed of (formate, acetate, propionate, oxalate, succinate, citrate-phosphate). Only for acetate, propionate and formate population of $H_{ox}c$ is observed. The dashed spectra represent HydA1 at pH 8 (100 mM Tris). The small peak at 2013 cm^{-1} indicates small contributions of H_{ox}-CO in some samples.

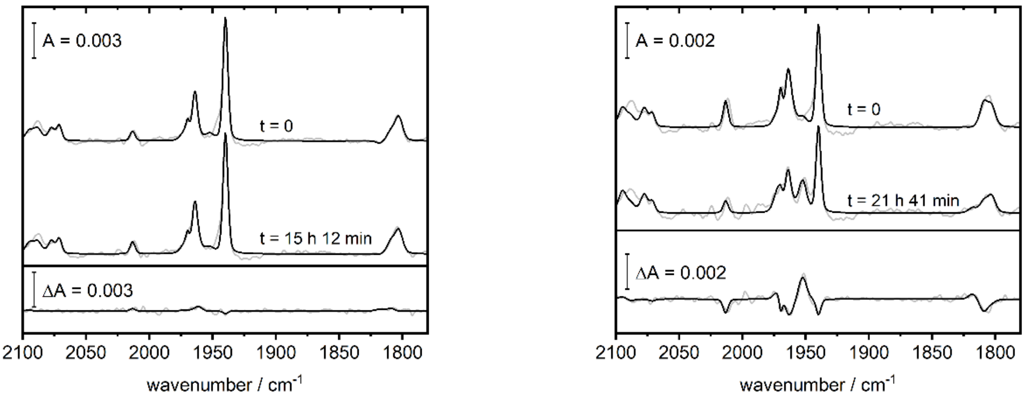

Figure S4 Absolute and difference ATR-FTIR spectra of HydA1 equilibrated with mixed buffer at pH 8 and pH 4. HydA1 exposed to pH 8 and pH 4 with the same buffer composed of 100 mM acetate and 100 mM Tris. The left panel shows the absolute spectra of HydA1 before (t=0) and after (15h 12min) exposure to mixed pH 8 buffer. The difference spectrum in the bottom shows that no $H_{ox}c$ species is formed. The right panel displays the same experiment conducted with mixed buffer at pH 4. The absolute and the difference spectrum show a clear population of $H_{ox}c$ (21 h 41 min). Grey lines represent the data and black lines the fit. Small changes of the peak at 2013 cm^{-1} indicates small contributions of H_{ox}-CO. The difference spectrum of the pH 4 sample shows a small depopulation of H_{ox}-CO after 21 hours.

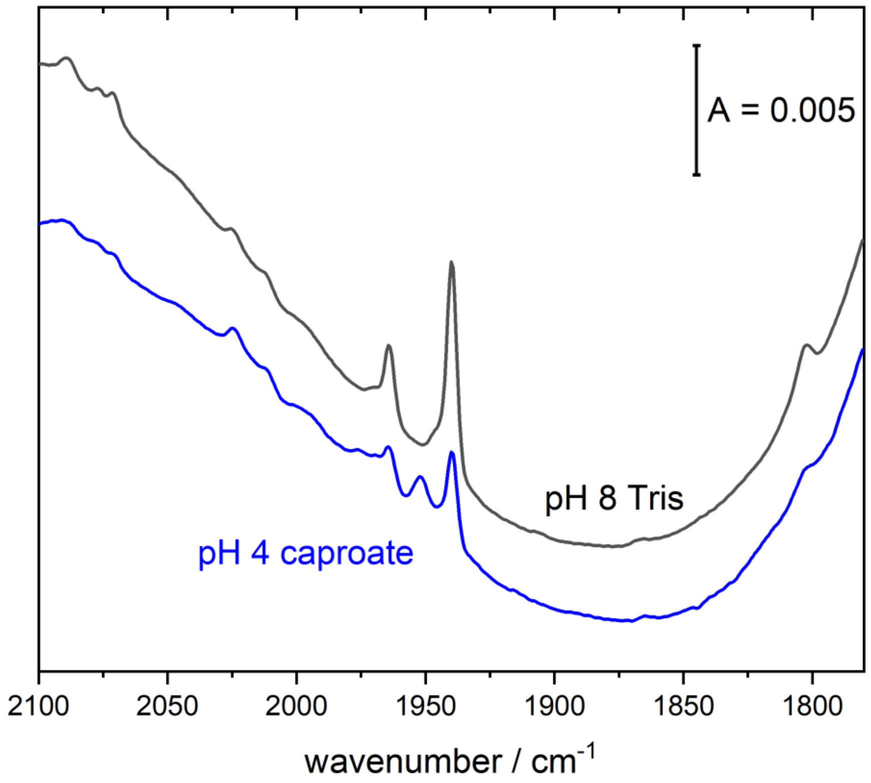

Figure S5 Absolute ATR-FTIR spectra of HydA1 equilibrated with mixed buffer at pH 8 (10 mM Tris buffer) and pH 4 with 100 mM capronate buffer. The spectrum in black shows the band pattern of H_{ox} as expected for pH 8. When the buffer is exchanged for capronate and adjusted to pH 4 a fraction of the H_{ox} population transfers into $H_{ox}c$ (blue spectrum). A band at 2013 cm^{-1} indicates small contributions of H_{ox}-CO. The subtraction of these two spectra resulted in the bottom difference spectrum in Fig.3 in the main text.

Supporting References:

1. D. W. Mulder, D. O. Ortillo, D. J. Gardenghi, A. V. Naumov, S. S. Ruebush, R. K. Szilagyi, B. Huynh, J. B. Broderick and J. W. Peters, *Biochemistry-Us*, 2009, **48**, 6240-6248.
2. L. S. Meszaros, B. Nemeth, C. Esmieu, P. Ceccaldi and G. Berggren, *Angew Chem Int Ed Engl*, 2018, **57**, 2596-2599.
3. S. T. Stripp, *Acs Catal*, 2021, **11**, 7845-7862.
4. M. Senger, S. Mebs, J. Duan, F. Wittkamp, U. P. Apfel, J. Heberle, M. Haumann and S. T. Stripp, *Proc Natl Acad Sci U S A*, 2016, **113**, 8454-8459.
5. M. Senger, S. Mebs, J. Duan, O. Shulenina, K. Laun, L. Kertess, F. Wittkamp, U. P. Apfel, T. Happe, M. Winkler, M. Haumann and S. T. Stripp, *Phys Chem Chem Phys*, 2018, **20**, 3128-3140.
6. D. W. Mulder, Y. Guo, M. W. Ratzloff and P. W. King, *J Am Chem Soc*, 2017, **139**, 83-86.
7. E. J. Reijerse, C. C. Pham, V. Pelmenschikov, R. Gilbert-Wilson, A. Adamska-Venkatesh, J. F. Siebel, L. B. Gee, Y. Yoda, K. Tamasaku, W. Lubitz, T. B. Rauchfuss and S. P. Cramer, *J Am Chem Soc*, 2017, **139**, 4306-4309.
8. M. Winkler, M. Senger, J. Duan, J. Esselborn, F. Wittkamp, E. Hofmann, U. P. Apfel, S. T. Stripp and T. Happe, *Nat Commun*, 2017, **8**, 16115.
9. D. W. Mulder, M. W. Ratzloff, E. M. Shepard, A. S. Byer, S. M. Noone, J. W. Peters, J. B. Broderick and P. W. King, *J Am Chem Soc*, 2013, **135**, 6921-6929.
10. D. W. Mulder, M. W. Ratzloff, M. Bruschi, C. Greco, E. Koonce, J. W. Peters and P. W. King, *J Am Chem Soc*, 2014, **136**, 15394-15402.
11. L. S. Meszaros, P. Ceccaldi, M. Lorenzi, H. J. Redman, E. Pfitzner, J. Heberle, M. Senger, S. T. Stripp and G. Berggren, *Chem Sci*, 2020, **11**, 4608-4617.
12. P. Knorzer, A. Silakov, C. E. Foster, F. A. Armstrong, W. Lubitz and T. Happe, *J Biol Chem*, 2012, **287**, 1489-1499.
13. M. W. Ratzloff, J. H. Artz, D. W. Mulder, R. T. Collins, T. E. Furtak and P. W. King, *J Am Chem Soc*, 2018, **140**, 7623-7628.
14. C. Lorent, S. Katz, J. Duan, C. J. Kulka, G. Caserta, C. Teutloff, S. Yadav, U. P. Apfel, M. Winkler, T. Happe, M. Horch and I. Zebger, *J Am Chem Soc*, 2020, **142**, 5493-5497.
15. V. Pelmenschikov, J. A. Birrell, C. C. Pham, N. Mishra, H. Wang, C. Sommer, E. Reijerse, C. P. Richers, K. Tamasaku, Y. Yoda, T. B. Rauchfuss, W. Lubitz and S. P. Cramer, *J Am Chem Soc*, 2017, **139**, 16894-16902.
